# Understanding medical travel from a source country perspective: a cross sectional study of the experiences of medical travelers from the Maldives

**DOI:** 10.1186/s12992-018-0375-4

**Published:** 2018-06-19

**Authors:** Mariyam Suzana, Helen Walls, Richard Smith, Johanna Hanefeld

**Affiliations:** 1 Department of Public Health, Faculty of Health Sciences, The Maldives National University, Haveeree Higun, Malé, 20-04 Republic of Maldives; 2 Department of Global Health and Development, London School of Hygiene & Tropical Medicine, Keppel street, London, WC1E 7HT UK

**Keywords:** Medical travel, Governance, Maldives

## Abstract

**Background:**

The resolution adopted in 2006 by the World Health Organization on international trade and health urges Member States to understand the implications of international trade and trade agreements for health and to address any challenges arising through policies and regulations. The government of Maldives is an importer of health services (with outgoing medical travelers), through offering a comprehensive universal health care package for its people that includes subsidized treatment abroad for services unavailable in the country. By the end of the first year of the scheme approximately US$11.6 m had been spent by the government of Maldives to treat patients abroad. In this study, affordability, continuity and quality of this care were assessed from the perspective of the medical traveler to provide recommendations for safer and more cost effective medical travel policy.

**Results:**

Despite universal health care, a substantial proportion of Maldivian travelers have not accessed the government subsidy, and a third reported not having sufficient funds for the treatment episode abroad. Among the five most visited hospitals in this study, none were JCI accredited at the time of the study period and only three from India had undergone the National Accreditation Board for Hospitals (NABH) in India. Satisfaction with treatment received was high amongst travelers but concern for the continuity of care was very high, and more than a third of the patients had experienced complications arising from the treatment overseas.

**Conclusion:**

Source countries can use their bargaining power in the trade of health services to offer a more comprehensive package for medical travelers. Source countries with largely public funded health systems need to ensure that medical travel is truly affordable and universal, with measures for quality control such as the use of accredited foreign hospitals to make it safer and to impose measures that ensure the continuity of care for travelers.

**Electronic supplementary material:**

The online version of this article (10.1186/s12992-018-0375-4) contains supplementary material, which is available to authorized users.

## Background

**Fig. 1:**
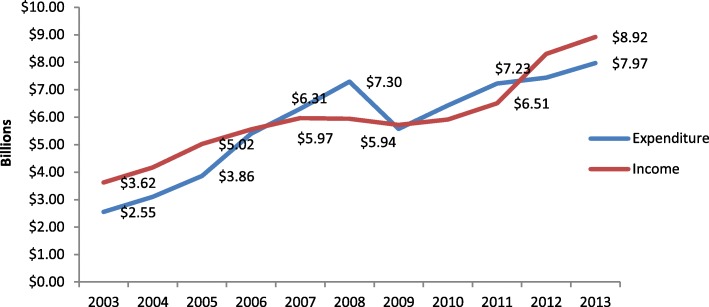
Income and expenditures from health related travel reported to IMF 2003 and 2013. Source: graphed by authors using IMF balance of payments data (2)

Consumption of health services abroad (or medical travel), the second mode of trade in health services defined in the World Trade Organization’s General Agreement on Trade in Services (GATS), is a growing but under reported phenomenon, with a rapidly expanding literature relating to this area of research [1]. While volume and scope of such travel are hard to estimate, countries do report to the International Monetary Fund (IMF) on income and expenditure relating to import and export of health services. According to health-related travel data reported to the IMF for 2013, 61 out of 188 member states have income from the export of health services (i.e.treating incoming medical travelers), amounting to US$ 8.9 billion in total. Seventy nine countries reported expenditure from the import of health services (outgoing medical travelers), which totaled US$7.9 billion [2]. IMF figures also show a 52% increase in the number of exporters and a 36% increase in the number of importing countries from 2003 data as shown in Fig. 1. While there are concerns relating to the reliability and comparability of these data, the figures point to a clear growth in trade in health services.

A resolution adopted in 2006 by the World Health Organization (WHO) on international trade and health (WHA 59.26) urges Member States to understand the implications of international trade and trade agreements for health and to address the issues through policies and regulations [3]. Research to date has included a focus on push and pull factors, including the motivations for medical travel, which identified that these involve a multiplicity of factors [4]. A review of Indonesian and Malaysian medical travelers, providers, intermediaries and policy makers showed that medical travel can influence the political and social system of the home country both at micro and macro levels by altering the perspectives, habits, expectations and accountability of the stakeholders in the home countries [5]. However, to date limited attention has been given to understanding medical travel from the viewpoint of the source countries.

Several reviews of the academic literature conducted over the past years (c.f. Hanefeld 2014 [1], Lunt N et al. 2016 [6]) have highlighted the need for survey based reliable estimates of size and scope of medical travel to understand better the effects of medical travel on country health systems and economies. This paper addresses this gap, through analysis of a new survey of medical travelers from the Maldives. It examines destinations, experiences and behaviour of medical travelers from the Maldives, both under a government funded scheme and amongst patients who pay for treatment out of pocket to explore push and pull factors. Based on this analysis, it makes recommendations on how to improve governance of medical travel to ensure patient safety. The paper contributes to a small body of knowledge that aims to improve governance mechanisms in medical travel.

### Introduction to the study setting

The Republic of Maldives, populated by 407,660 people [7], is an import oriented small island state in the Indian Ocean and one of the top importers of health care in the South Asian region. Health related travel data from the IMF shows that on average the Maldives spent US$65 million annually for the past 10 years on importing health care [2]. According to the Balance of Payments manual of the IMF, the health related travel component covers all goods and services related to health purchased from an economy by travelers. That is, it includes all medical equipment, medical consumables and out-of-pocket purchases. Due to the geographic dispersion of the islands, travel is common and a recent study by the Ministry of Tourism of Maldives showed that domestic travel between the islands alone incurred Maldivian Rufiyaa (MRF) 600 m (approximately US$ 39.8 m) annually in 2015, out of which 66% of the travel was for medical purposes [8]. The Maldivian government introduced a policy of Universal Health Care (UHC) ‘Aasandha’ in 2012, a comprehensive benefit package that includes subsidized treatment abroad for services unavailable in the country. By the end of the first year of UHC, a total of 276,033 citizens (84% of the population) had utilized the scheme at least once and MRF 180 million (approximately US$11.6 million) was spent by the government of Maldives to treat patients abroad [9]. Within the $65 m reported by the IMF, the $11.6 m relates directly to government expenditure to treat the subsidized travelers.

The Maldivian government holds strong bilateral relationships with neighbouring India and Sri Lanka and is a signatory to the South Asian Association for Regional Corporation (SAARC), an organization established among seven countries of the region to promote the welfare of its people. The public tertiary hospital on the capital island of the Maldives is a gift from India in 1995 [10]. The government of the Maldives funds medical treatment overseas from contracted providers in India and Sri Lanka through the public referral scheme ‘Aasandha’. Under the scheme, public sector physicians can prescribe for patients to travel abroad for a specific procedure, and patients are free to choose from a list of pre-approved providers in India and Sri Lanka. The scheme is managed by a public limited company which pays the providers directly for treatment received by the patients and manages the airline tickets for patients to travel abroad. Overseas hospitals are enlisted into the scheme under a signed memorandum of understanding. Hospitals with local or international accreditations are selected for service provision. However, the scheme does not extend to travel, accommodation, or food in the destination. Only 0.18% of all the transactions of Aasandha in its first year of implementation were found to have required out of pocket expenditure on medical costs [9] which would have resulted in travelers needing to seek alternative sources of funds to cope with treatment costs abroad. In addition to the government program, a large number of citizens who choose to pay out of pocket for their treatment abroad make their own choice of destinations and hospitals [11].

This study has involved analyses of survey data from 815 medical travelers from the Maldives. Initial findings of the demographic and overseas health care utilization characteristics of which have been reported elsewhere (refer to [11]). This paper presents additional findings from a semi structured questionnaire administered to a subset of 120 travelers to examine their experiences during treatment in India and Sri Lanka in more detail. As the study was conducted in a predominantly publicly funded health system, we focused on affordability, continuity and quality of care from the perspective of the medical traveler, and provide recommendations for safer and more cost effective medical travel policy.

## Methods

### Study design

Analysis of data from a cross sectional survey of 815 medical travelers from the Republic of Maldives who travelled overseas during June to December 2013. The design of this cross-sectional study has been described elsewhere (refer to [11]). Refusals to participate were negligible. In addition a subset of travelers from the same sample were selected based on residence (Haadhaal Atoll, Kaafu Atoll, Seenu Atoll and Gnaviyani Atoll). This subset received an additional semi structured questionnaire to which 120 out of 379 patients responded.

### Selection of participants

The study was restricted to Maldivian travelers who had returned from medical treatment abroad. Participants were stratified by their use (or not) of the government subsidy for travel. The subset represented 30 travelers from Haadhaal atoll, 49 from Kaafu atoll, 28 from Seenu atoll and 13 travelers from Gnaviyani atoll who were interviewed using an 18 item semi structured questionnaire which focused on the experiences, expectations, and satisfaction of the last medical travel episode. The subset sample comprised the most populated four atolls of the Maldives and includes both subsidized and OOP travelers. In the Maldivian context, although all citizens are eligible to apply for the subsidy, some refrain from applying for it. These groups of people pay out of pocket even when they go overseas for treatment.

### Variables

Questionnaire 1 (administered to all study participants) collected data on demographic characteristics, utilization of overseas health care and costs of health care abroad. The semi structured questionnaire collected data on the experiences and expectations of the travelers. It focused on whether travelers experienced complications, travel discomfort, any form of exploitation, language barriers. It collected prior information about the provider, expressed concern about hospital infections, competence of the health personnel, whether informed consent was taken before procedures and drugs were monitored, whether severity of the disease could have been prevented with care from home country, concern for continuity of care and expectation to have the service available in the home country. Monthly household income was used to show the living standard measure. The destination variable showed one case that travelled to Italy and five cases to Thailand. These six cases were omitted to enable proper statistical analysis.

### Statistical analysis

Descriptive analysis was performed using the open source R software, version 3.1.0. [12]. Bivariate analysis was used to compare between destinations and between subsidized and non-subsidized travelers using chi squared test, Fisher’s exact test and Ranksum tests.

### Ethical considerations

Ethical approval for the study was sought by the authors’ institutions.

## Results

Providers and destinations frequented by Maldivian medical travelers: Among the total sample (*n* = 815), the majority of the travelers (66.9%) visited Indian hospitals for treatment. Across Indian providers, the most highly frequented hospitals were the KIMS hospital, Ananthapuri hospital, NIMS hospital and RCC. In Sri Lanka, the most visited were Lanka Hospital, Nawaloka hospital and Asiri Surgical Hospital. A high proportion of travelers (29.6%) visited other hospitals in both India and Sri Lanka, which fall out of the contracted list of hospitals with the Maldivian government in these countries. (refer to Additional file 1: Table S1 (*N* = 809).

Choice of destination by financial protection to travel and disease profile: Among the total sample (*n* = 815), we analyzed the conditions for which patients travel by the destination to which they travelled (refer to Additional file 2: Table S2). A slightly wider range of treatments were sought in India than Sri Lanka (20 vs 18 disease groups). The subsidized patients had been treated in India mostly for external causes (V01-Y98) (17%), neoplasms (C00-D48) (16%) and circulatory diseases (I00–99) (13%) and in Sri Lanka for services related to the status of their health which includes routine health checkups (Z00–99) (24%) and circulatory diseases (I00–99) (16%). This differed in profile to the out of pocket payers who have sought treatment in India mostly for injuries (S00-T98) (16%) and Sri Lanka for services relating to the status of their health (Z00–99) (32%).

The subsequent results describe the demographics of the subset of medical travelers from whom the experiences, expectations and the coping mechanism during a medical travel episode were derived.

There were no statistically significant differences in demographics, utilization of health care, and in medical and non-medical costs between the two destinations in the subset of travelers from the four most populated atolls of the Maldives. However, it is evident that none of the medical travelers lived below the poverty line ($2 per day) (Table 1).

**Table 1:** Demographic characteristics of medical travelers by destination, N = 120

	India	Sri Lanka	Total	*P* value
Total	80	39	120	
Gender				0.94
Female	41 (51.2)	21 (53.8)	62 (52.1)	
Male	39 (48.8)	18 (46.2)	57 (47.9)	
Age group				0.06
Children < 9 yrs	13 (16.2)	9 (23.1)	22 (18.5)	
Adolescents 10-19 yrs	6 (7.5)	1 (2.6)	7 (5.9)	
Youth 20-29 yrs	11 (13.8)	0 (0)	11 (9.2)	
Adult 30-59 yrs	41 (51.2)	25 (64.1)	66 (55.5)	
Elderly > 60 yrs	9 (11.2)	4 (10.3)	13 (10.9)	
Region of residence				0.45
Haadhaal Atoll	22 (27.5)	8 (20.5)	30 (25.2)	
Kaafu Atoll	29 (36.2)	20 (51.3)	49 (41.2)	
Seenu Atoll	19 (23.8)	8 (20.5)	27 (22.7)	
Gnaviyani Atoll	10 (12.5)	3 (7.7)	13 (10.9)	
Occupation				0.95
Civil servant	19 (23.8)	11 (28.2)	30 (25.2)	
Private sector	6 (7.5)	3 (7.7)	9 (7.6)	
Own business	15 (18.8)	8 (20.5)	23 (19.3)	
Not employed	24 (30)	9 (23.1)	33 (27.7)	
Other	16 (20)	8 (20.5)	24 (20.2)	
Financial protection for overseas treatment				1
Government and Private	37 (46.2)	18 (46.2)	55 (46.2)	
Private finance	43 (53.8)	21 (53.8)	64 (53.8)	
Monthly household income				0.46
$61–$1305	38 (47.5)	15 (38.5)	53 (44.5)	
> = $1306	42 (52.5)	24 (61.5)	66 (55.5)	
Length of stay				0.06
<=1 week	21 (26.2)	5 (12.8)	26 (21.8)	
2 weeks	26 (32.5)	21 (53.8)	47 (39.5)	
3 weeks	19 (23.8)	4 (10.3)	23 (19.3)	
1 month	4 (5)	1 (2.6)	5 (4.2)	
> 1 month	10 (12.5)	8 (20.5)	18 (15.1)	
Motivation for travel				0.72
quality of care	15 (18.8)	8 (20.5)	23 (19.3)	
unavailability of service	45 (56.2)	21 (53.8)	66 (55.5)	
continuity of care	4 (5)	1 (2.6)	5 (4.2)	
better prices	0 (0)	1 (2.6)	1 (0.8)	
long waiting time	5 (6.2)	1 (2.6)	6 (5)	
other	11 (13.8)	7 (17.9)	18 (15.1)	
Medical cost ($)				0.89
median(IQR)	670.4 (335.2,1910.7)	694.6 (272.2,1467.6)	682.5 (335.2,1877.2)	
Non-medical cost ($)				0.18
median(IQR)	2449.8 (2105.2,3336.4)	2695.1 (2172.9,4597.2)	2545 (2112.5,3798.7)	

Among the subset of travelers (Table 2), more than 90% of the medical travelers were satisfied with their last treatment episode with no significant difference by financial protection for travel by destination. Concern for the continuity of care was very high (87.5%), and most people expected the treatment to be available in the home country (84%). Expression of concern for continuity of care was significantly higher among the government subsidized patients. More than a third of the patients had experienced complications arising from the treatment overseas and more than half had faced problems with communication. Half of the respondents did not believe that the government subsidy was given to the neediest (55.8%) and this was significantly higher amongst the out of pocket payers. A quarter of the patients had experienced financial problems relating to the medical travel (26.7%).

**Table 2:** Experiences and expectations of the medical traveler by financial protection for travel

Experiences and expectations	Total	Subsidized	Non subsidized	P value
	N = 120	N = 55	N = 65	
Overall satisfaction				0.752
Yes	110 (91.7)	51 (92.7)	59 (90.8)	
No	10 (8.3)	4 (7.3)	6 (9.2)	
Misinformation or exploitation				0.323
Yes	16 (13.3)	5 (9.1)	11 (16.9)	
No	104 (86.7)	50 (90.9)	54 (83.1)	
Travel discomfort				0.492
Yes	24 (20)	9 (16.4)	15 (23.1)	
No	96 (80)	46 (83.6)	50 (76.9)	
Prior information about the hospital				0.529
Yes	94 (78.3)	45 (81.8)	49 (75.4)	
No	26 (21.7)	10 (18.2)	16 (24.6)	
Consent taken before giving drugs				0.096
Yes	106 (88.3)	52 (94.5)	54 (83.1)	
No	14 (11.7)	3 (5.5)	11 (16.9)	
Consent taken before medical procedure				0.096
Yes	106 (88.3)	52 (94.5)	54 (83.1)	
No	14 (11.7)	3 (5.5)	11 (16.9)	
Consent taken before giving therapy				0.274
Yes	106 (88.3)	51 (92.7)	55 (84.6)	
No	14 (11.7)	4 (7.3)	10 (15.4)	
Concern for hospital Infection				0.281
Yes	33 (27.5)	12 (21.8)	21 (32.3)	
No	87 (72.5)	43 (78.2)	44 (67.7)	
Doctor’s competence				0.505
Yes	111 (92.5)	52 (94.5)	59 (90.8)	
No	9 (7.5)	3 (5.5)	6 (9.2)	
Nurse competence				0.541
Yes	108 (90)	51 (92.7)	57 (87.7)	
No	12 (10)	4 (7.3)	8 (12.3)	
Staff competence				1
Yes	111 (92.5)	51 (92.7)	60 (92.3)	
No	9 (7.5)	4 (7.3)	5 (7.7)	
Experience of complications				0.237
Yes	45 (37.5)	17 (30.9)	28 (43.1)	
No	75 (62.5)	38 (69.1)	37 (56.9)	
Language barrier				1
Yes	83 (69.2)	38 (69.1)	45 (69.2)	
No	37 (30.8)	17 (30.9)	20 (30.8)	
Concern for continuity of care				0.015
Yes	105 (87.5)	53 (96.4)	52 (80)	
No	15 (12.5)	2 (3.6)	13 (20)	
Expectation for availability of the service in home country				1
Yes	101 (84.2)	46 (83.6)	55 (84.6)	
No	19 (15.8)	9 (16.4)	10 (15.4)	
Could prevention measures in home country have reduced the severity of your disease?				0.575
Yes	72 (60)	31 (56.4)	41 (63.1)	
No	48 (40)	24 (43.6)	24 (36.9)	
Was money sufficient?				0.73
Yes	88 (73.3)	39 (70.9)	49 (75.4)	
No	32 (26.7)	16 (29.1)	16 (24.6)	
Relevance of the beneficiaries of subsidies				0.002
Yes	53 (44.2)	33 (60)	20 (30.8)	
No	67 (55.8)	22 (40)	45 (69.2)	

**Fig. 2:**
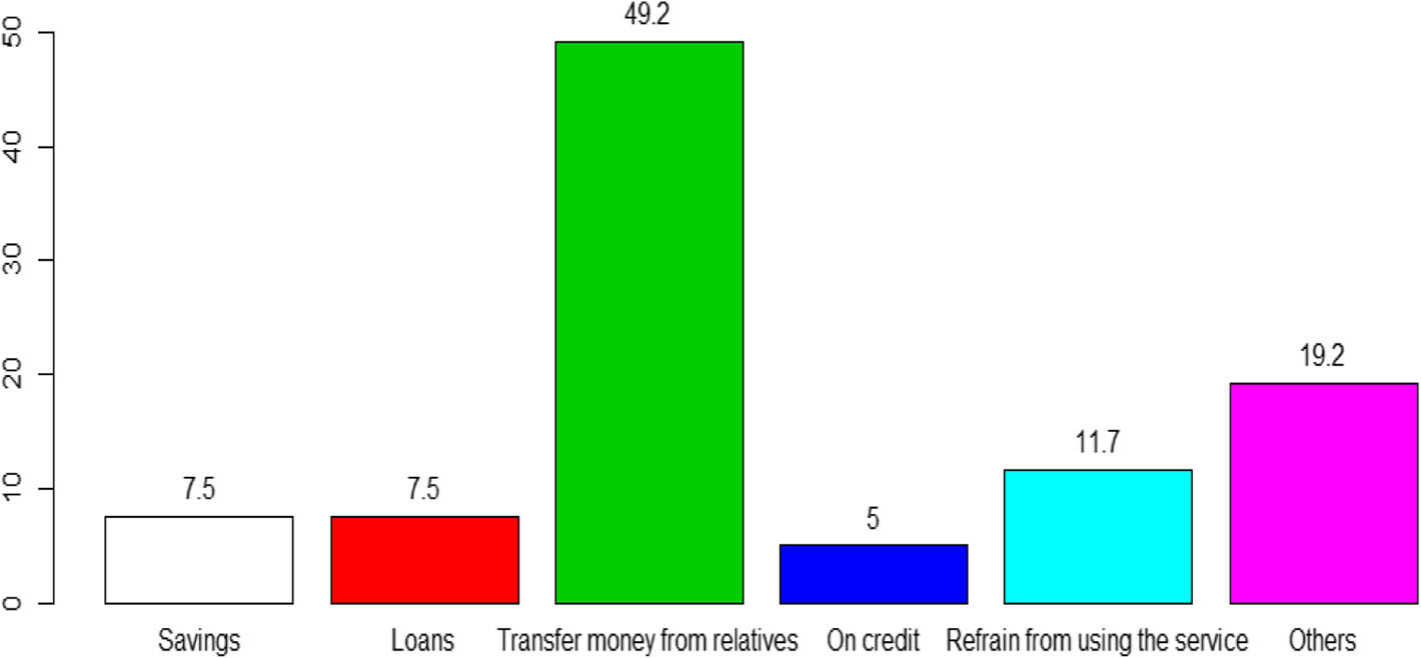
Coping mechanisms among medical travelers, *N* = 120

In both destinations, the most common coping mechanism reported by travelers was the transfer of money from relatives in the home country (49.2%) when money taken for the treatment became insufficient (Fig. 2). Use of savings was reportedly higher among travelers in Sri Lanka (12.8% vs 5% in India) 15% in India reported that they would refrain from acquiring the treatment while this percentage was lower in Sri Lanka (data not shown).

We also examined travelers’ views on how the government could further assist travelers. Among the subset of 120 travelers, 73.2% responded to this open ended question. Making UHC more accessible for medical travelers, visa facilitation and appeal for services to be made available in the home country were the three most common views of Maldivian medical travelers going to India and Sri Lanka.

## Discussion

This study has described the most highly frequented medical travel destinations, the disease profiles and the motivations of medical travelers from a source country perspective. Maldivian medical travelers travelled both for critical medical procedures such as neoplasms and simple ones such as routine health checkups. The subsidized patients were treated in India mostly for illnesses due to external (environmental) causes and neoplasms and in Sri Lanka for services related to the factors influencing the status of their health (described as circumstances other than a disease or an injury) and for circulatory diseases. The out of pocket payers sought India mostly for injuries and Sri Lanka for services relating to factors influencing the status of their health. Travelers were found to be highly satisfied with their treatment episodes abroad. However, expectations to have the services available in the home country were as high.

India and Sri Lanka were by far the most frequented medical travel destinations among the Maldivians. Our findings follow the pattern of intra-regional medical travel observed among countries such as Myanmar [13], Cambodia and Laos [14] into neighbouring Thailand, between the USA, the Caribbean, Central and South American countries [15] and inflow to India from SAARC countries and Commonwealth states in 2014 [16]. It may reflect the cultural similarities and political relations between and among these countries. Doctors and nurses from India, Sri Lanka and neighbouring countries make up the majority of the expatriate doctor and nurse workforce in the Maldivian public health facilities, resulting in them being a familiar choice in the decision making process of travelers to choose India and Sri Lanka. In addition, the arrangement for attaining a medical visa to India and offer of public subsidy to get treated in these two countries contributes to the popularity of these two countries for medical travel.

**Evidence on affordability,** a major tenet of the Asandha policy is mixed. Despite universal health care, a substantial proportion of travelers have declined the government subsidy, and findings also point towards the fact that the poor (people living below $2 per day poverty line) have not accessed medical travel. In addition, a third of participants reported not having sufficient funds for the treatment episode abroad. There is existing evidence of differential use of health services by the poor [17, 18], due to stigma [19], transportation costs [20], while higher levels of ill health is existent among the poor. Further research is needed to identify the reasons for the lack of access to the UHC program involving medical travel abroad by the poor.

A major coping mechanism among medical travelers was found to be money transfers from the relatives in the home country, which highlights the importance for medical travelers of easier banking facilities between the destination countries and the source country. According to the household income and expenditure survey of 2010, more than one million US dollars were transferred abroad by Maldivian households for medical purposes [21]. Against this financial burden, the views of medical travelers to increase access to the universal coverage program ‘Aasandha’ and the call to make services available in the domestic health system are reasonable. In a study by Noor (nd) [22], Bangladeshis also preferred improvement to the home country health system through foreign direct investments, private sector involvement, increasing budgetary allocations for existing health facilities and investment in medical education.

### Evidence on continuity of care

Concern for the continuity of care was very high. More than a third of the patients had experienced complications arising from the treatment overseas and two thirds had faced problems with communication. Herrick (2007) found that patients whose local GPs may not have been willing to provide services for particular complications were more likely to travel back overseas as the cost of treating unexpected complications was lower abroad. A study by Hanefeld et al. (2013) [23] found that associated costs to the local health system, in this case of the UK, were an estimated 8.2million pounds annually from medical tourism for cosmetic purposes. Where patients travel for a variety of medical treatments, appropriate preventive care measures in the home country can help to reduce the burden of medical travel.

In this study, the medical travelers from the Maldives were driven to seek care abroad due to the unavailability of health services in the home country and for quality healthcare. This contrasts with patients from the USA and Europe who reportedly travel to Asia in search of low cost treatments [24], whilst wait times were found to be the most significant driver of Canadian involvement in medical tourism [25]. Medical treatment overseas may be the solution for health services that are not cost effective to be provided in small populations such as that of the Maldives. For these services, a more comprehensive package that includes better hospital pricing, accommodation, food, travel arrangements and continuity of care needs to be negotiated with the hospitals, airlines, estate agents and the governments involved. This is especially important when the Maldivian government has the purchasing power of 78,000 subsidized patients [11] a year who travel to medical travel destinations. Alternatively, the package could be sold to the bidding country that provides the best offer.

### Evidence on quality of health services

Many studies have shown that hospital accreditation has a positive impact on quality of health services [26, 27] and is used as a marketing tool, while other studies have given mixed evidence of accreditation impact when the size and activities of the hospital were taken into account [28]. We found that among the five most visited Sri Lankan and Indian hospitals in this study, none were JCI accredited during the period of study and only three from India had undergone national accreditation by the National Accreditation Board for Hospitals (NABH) in India. The lack of a global body for accreditation of hospitals such as that by UN or WHO noted by Lunt and colleagues 2011 [29] may explain the absence of accreditation among providers in this study too. Furthermore, the preference to visit other hospitals that are not under contract with the government of Maldives may indicate unavailability of the treatment at the contracted hospitals, the offer of cheaper prices, or travelers may have chosen their hospital based on the location of a certain practitioner. Study findings highlight the need for better screening of foreign providers and their services for the protection and safety of medical travelers from middle income countries (MICs) to MICs.

A very high percentage of the Maldivian medical travelers were satisfied with their last treatment episode. Studies have shown an association between perceived health service quality and intention to revisit [30, 31]. Contrary to this, Maldivian travelers were satisfied with their treatment episode overseas, but their expectation for these services to be available in the home country was also high. This suggests that medical tourism should be a choice, not a necessity.

Most of the Maldivian patients in this study had acquired information about the hospital before travelling (78.3%) and the occurrence of misinformation or exploitation was low amongst travelers. A high literacy rate at 97.7% and high prevalence of mobile telephone use (194.6 mobile subscriptions per 100 person in 2015) among Maldivians [7, 32] may have contributed to the informed travelling among Maldivians. However, the sources of this information and its credibility are unknown. Patients have been found to be more receptive to information provided by public sources [33], suggesting that government initiated research and analysis of destinations, providers, services and costs of treatment will support improvements to the safety of medical travelers.

#### Study limitations

Include possible bias arising from the low response rate (32%) among the subset of travelers participating in the study, and by the fact that the self-reported responses in the two surveys cannot be validated by other sources. In addition, characteristics of the travelers who refused were not collected, which limits the researchers from identifying the potential effect of their lack of inclusion in the study on the study results. Despite the limitations, the study represents analysis of one of the largest surveys conducted among medical travelers from a source country, and the findings fit within the larger global context of medical travel experience.

## Conclusions

Medical travel from the Maldives was found to be driven by shortages in the local health system and the desire for better quality of health care than was available in the source country. Maldivian medical travelers have sought India and Sri Lanka as a solution for shortages in the domestic system. The fact that most of overseas hospitals enlisted under the government were from India and Sri Lanka and the geographic proximity may have contributed to this decision. Treatments ranged from highly critical diseases to routine health checkups. This study highlighted three main areas for policy interventions; affordability equity, continuity of care and quality of care for medical travelers.

With a bargaining power of more than 78,000 patients annually [11], a more comprehensive package that includes better hospital pricing, accommodation, food, and travel arrangements could be negotiated by the Maldivian government with the foreign hospitals, airlines, estate agents and other governments involved. In public funded health systems, government financing of such public private partnerships can help make medical travel affordable for those in most need.

Satisfaction with treatment received was high amongst travelers but concern for the continuity of care was very high, and more than a third of the patients had experienced complications arising from the treatment overseas. As the patients had travelled for a wide variety of medical treatments, establishing cost effective measures of preventive and follow up care at the home country may help to reduce medical travel.

Among the five most visited hospitals in this study, none were JCI accredited at the time of the study period and only three from India had undergone national accreditation by the National Accreditation Board for Hospitals (NABH) in India. The findings highlight the need for government initiated screening of providers, and monitoring and evaluation of their services and success rates, to support better Maldivian patients and doctors to make informed healthcare decisions.

We recommend introducing measures to ensure more equitable access to the universal health care programme to make medical travel affordable and truly universal, to implement measures that ensure the continuity of care for travelers and measures for quality control and safety such as the usage of accredited foreign hospitals. Findings from this study contribute to understanding better the areas in need of better governance in the medical travel industry which can be achieved through bilateral and regional coordination of medical travel.

## Additional files

Additional file 1: **Table S1.** Providers and destinations frequented by Maldivian medical travelers, 2013. (DOCX 17 kb)

Additional file 2: **Table S2.** Choice of destination by financial protection to travel and disease profile (DOCX 17 kb)

## Abbreviations

GATS: General Agreement on Trade in Services
IMF: International Monetary Fund
JCI: Joint Commission International
MIC: Middle income country
MRF: Maldivian Rufiyaa
NABH: National accreditation Board for hospitals
SAARC: South Asian Association for Regional Corporation
UHC: Universal Health Care
WHA: World Health Assembly
WHO: World Health Organization
