# Supplementary material for: Understanding medical travel from a source country perspective: a cross sectional study of the experiences of medical travelers from the Maldives
Source: Global Health. 2018 Jun 19;14:58. doi: 10.1186/s12992-018-0375-4 (PMC6010160; doi:10.1186/s12992-018-0375-4)
Supplement: Supplementary file 1 — Table S1. Providers and destinations frequented by Maldivian medical travelers, 2013. (DOCX 17 kb) [file 12992_2018_375_MOESM1_ESM.docx]

Additional file 1: **Table S1**. Providers and destinations frequented by Maldivian medical travelers, 2013

| **Name of hospital** | **Total** | |
| --- | --- | --- |
|  | ***N*** | ***%*** |
| **India** |  |  |
| •KIMS Hospital | 101 | 12.5% |
| •Ananthapuri Hospital | 67 | 8.3% |
| •NIMS hospital | 37 | 4.6% |
| •Regional Cancer Center | 34 | 4.2% |
| •Chaithanya Eye Hospital | 22 | 2.7% |
| •Amrita Hospital (AIMS) | 18 | 2.2% |
| Lords Hospital | 17 | 2.1% |
| •Cosmopolitan Hospital | 11 | 1.4% |
| *Apollo Hospital | 9 | 1.1% |
| •Ganga medical centre and hospital | 9 | 1.1% |
| Vasan Eye care | 9 | 1.1% |
| Nova Medical Center | 3 | 0.4% |
| Srichitra Medical Centre | 3 | 0.4% |
| Arvindh eye hospital | 2 | 0.2% |
| GKNM Hospital | 2 | 0.2% |
| KMCHospital | 2 | 0.2% |
| PRS Hospital | 2 | 0.2% |
| GG Hospital | 1 | 0.1% |
| •Global Hospitals | 1 | 0.1% |
| Metropolis healthcare | 1 | 0.1% |
| Prudence Hospital | 1 | 0.1% |
| St Joseph Hospital | 1 | 0.1% |
| Others | 188 | 23.2% |
| **Sri Lanka** |  |  |
| *•Lanka Hospital | 123 | 15.2% |
| •Nawaloka Hospital | 53 | 6.6% |
| •Asiri Surgical Hospital | 13 | 1.6% |
| Central Hospital | 11 | 1.4% |
| *Durdans Hospital | 9 | 1.1% |
| Apollo Hospital | 3 | 0.4% |
| Western Infirmary Hospital | 1 | 0.1% |
| Park Hospital | 1 | 0.1% |
| •Hemas Hospital | 1 | 0.1% |
| Golden Key Eye and ENT Hospital | 1 | 0.1% |
| Others | 52 | 6.4% |
| **Total** | **809** | 100.0% |
| *JCI accredited in 2006, 2014 and 2014 in ascending order ([13](#_ENREF_13)), •under contract with the government of Maldives ([14](#_ENREF_14)) | | |
