# Supplementary material for: Understanding medical travel from a source country perspective: a cross sectional study of the experiences of medical travelers from the Maldives
Source: Global Health. 2018 Jun 19;14:58. doi: 10.1186/s12992-018-0375-4 (PMC6010160; doi:10.1186/s12992-018-0375-4)
Supplement: Supplementary file 2 — Table S2. Choice of destination by financial protection to travel and disease profile (DOCX 17 kb) [file 12992_2018_375_MOESM2_ESM.docx]

Additional file 2: **Table S2**. Choice of destination by financial protection to travel and disease profile

| **Disease group (ICD 10)** | **India** | | |  | **Sri Lanka** | | |  |
| --- | --- | --- | --- | --- | --- | --- | --- | --- |
|  | **Subsidized** | | **Non subsidized** | | **Subsidized** | | **Non subsidized** | |
|  | **N** | **%** | **N** | **%** | **N** | **%** | **N** | **%** |
| Infectious (A00-B99) | 1 | 0% | 13 | 4% | 2 | 2% | 11 | 7% |
| Neoplasms (C00-D48) | 38 | 16% | 6 | 2% | 6 | 6% | 1 | 1% |
| Blood (D50-D89) | 2 | 1% | 1 | 0% | 3 | 3% | 0 | 0% |
| Endocrine (E00-E90) | 8 | 3% | 25 | 8% | 0 | 0% | 1 | 1% |
| Mental (F00-F99) | 4 | 2% | 9 | 3% | 0 | 0% | 3 | 2% |
| Nervous (G00-G99) | 19 | 8% | 33 | 11% | 4 | 4% | 13 | 8% |
| Eye (H00-H59) | 22 | 9% | 18 | 6% | 6 | 6% | 4 | 3% |
| Ear (H60-H95) | 4 | 2% | 1 | 0% | 0 | 0% | 0 | 0% |
| Circulatory (I00-I99) | 30 | 13% | 28 | 9% | 17 | 16% | 7 | 4% |
| Respiratory (J00-J99) | 3 | 1% | 5 | 2% | 5 | 5% | 12 | 8% |
| Digestive (K00-K93) | 9 | 4% | 16 | 5% | 5 | 5% | 5 | 3% |
| Skin (L00-L99) | 1 | 0% | 1 | 0% | 0 | 0% | 0 | 0% |
| Musculoskeletal (M00-M99) | 5 | 2% | 34 | 11% | 5 | 5% | 21 | 13% |
| Genitourinary (N00-N99) | 21 | 9% | 20 | 7% | 13 | 12% | 8 | 5% |
| Pregnancy (O00-O99) | 1 | 0% | 2 | 1% | 0 | 0% | 0 | 0% |
| Perinatal (P00-P96) | 7 | 3% | 1 | 0% | 2 | 2% | 0 | 0% |
| Congenital malformations (Q00-Q99) | 7 | 3% | 19 | 6% | 0 | 0% | 1 | 1% |
| Symptoms & signs (R00-R99) | 13 | 5% | 23 | 8% | 7 | 7% | 16 | 10% |
| Injuries (S00-T98) | 4 | 2% | 50 | 16% | 2 | 2% | 5 | 3% |
| External causes (V00-Y98)° | 40 | 17% | 0 | 0% | 3 | 3% | 1 | 1% |
| *Health status (Z00-Z99) | 0 | 0% | 0 | 0% | 25 | 24% | 51 | 32% |
| **Total (809 cases)** | **239** | **100%** | **305** | **100%** | **105** | **100%** | **160** | **100%** |
| *occasions when circumstances other than a disease, injury or external cause classifiable to any other category | | | | | | | | |
| °Environmental events and circumstances as the cause of injury, poisoning and other adverse effects | | | | | | | | |
